# Evaluation of MTBDR*plus* and MTBDR*sl* in Detecting Drug-Resistant Tuberculosis in a Chinese Population

**DOI:** 10.1155/2016/2064765

**Published:** 2016-07-25

**Authors:** Wei Lu, Yan Feng, Jianming Wang, Limei Zhu

**Affiliations:** ^1^Department of Chronic Communicable Diseases, Center for Disease Control and Prevention of Jiangsu Province, Nanjing 210009, China; ^2^Department of Epidemiology, School of Public Health, Nanjing Medical University, Nanjing 211166, China; ^3^Division of Parasite Disease Intervention, Zhejiang Provincial Center for Disease Control and Prevention, Zhejiang 310051, China; ^4^The Innovation Center for Social Risk Governance in Health, Nanjing 211166, China

## Abstract

*Background*. This study aims to evaluate GenoType MTBDR*plus* and GenoType MTBDR*sl *for their ability to detect drug-resistant tuberculosis in a Chinese population.* Methods*. We collected 112* Mycobacteria tuberculosis *strains from Jiangsu province, China. The conventional DST and line probe assay were used to detect drug resistance to rifampicin (RFP), isoniazid (INH), ofloxacin (OFX), kanamycin (Km), and ethambutol (EMB).* Results*. The sensitivity and specificity were 100% and 50% for RFP and 86.11% and 47.06% for INH, respectively. The most common mutations observed in MTBDR*plus *were* rpoB*WT8 omission + MUT3 presence,* katG*WT omission + MUT1 presence, and* inhA*WT1 omission + MUT1 presence. For drug resistance to OFX, Km, and EMB, the sensitivity of MTBDR*sl* was 94.74%, 62.50%, and 58.82%, respectively, while the specificity was 92.59%, 98.81%, and 91.67%, respectively. The most common mutations were* gyrA*WT3 omission + MUT3C presence,* rrs*MUT1 presence,* embB*WT omission + MUT1B presence, and* embB*WT omission + MUT1A presence. Sequencing analysis found several uncommon mutations.* Conclusion*. In combination with DST, application of the GenoType MTBDR*plus* and GenoType MTBDR*sl* assays might be a useful additional tool to allow for the rapid and safe diagnosis of drug resistance to RFP and OFX.

## 1. Introduction

Antimicrobial resistance (AMR) is the ability of a microorganism to resist an antimicrobial medicine to which it was originally sensitive [1]. Resistant microorganisms are able to withstand attacks by these medicines, resulting in treatment failure, a prolonged disease process, and increased risks of microorganisms spreading. AMR is detrimental to the successful control of infectious diseases, and it increases the economic burden on individuals and societies. Recently, the emergence of drug-resistant tuberculosis (TB) has drawn greater attention to AMR. The discovery of MDR (at least resistance to isoniazid [INH] and rifampicin [RFP]) and XDR (resistance to INH, RFP, and any fluoroquinolones [FLQs] and to one of amikacin [AM], capreomycin [CAP], and kanamycin [Km]) has posed a difficult challenge for TB control [2, 3].

According to the global tuberculosis report in 2013, 450,000 people developed MDR-TB, and 170,000 die of MDR-TB annually, with the highest levels in eastern Europe and central Asia [4]. Current assays for detecting drug-resistant tuberculosis include conventional drug susceptibility testing (DST), molecular-based DST, sequencing of known genetic loci, line-probe assay, and GeneXpert MTB/RIF. Conventional DST remains a common choice in many countries, especially in source-limited and funding-lacking areas, and it is the only gold standard when evaluating new molecular techniques [5]. However, it is constrained by the slow growth characteristics of* M. tuberculosis*, which can take two to four weeks on solid culture medium [6]. In addition, poor standardization of conventional DST persists, including inoculum size, dispersion of bacillary clumps, subculturing bias, testing environment (temperature and PH), and critical concentrations of certain drugs [5].

Because the genetic mutations in the* M. tuberculosis* genome were proved to be associated with the phenotype of drug resistance [7, 8], molecular techniques have made the rapid detection of MDR or XDR based on these mutations possible. The WHO has recommended two molecular methods: line probe assays (LPAs) [9] and Xpert MTB/RIF [10]. Compared with Xpert MTB/RIF, LPAs are able to detect resistance to other drugs in addition to RFP using hybridization assays. Moreover, LPAs can detect heteroresistance, which is defined as the coexistence of susceptible and resistant bacteria in the same specimen [11, 12]. This type of heteroresistance is difficult to identify using conventional DST [13].

GenoType MTBDR*plus* and GenoType MTBDR*sl* are two commercial versions of LPAs designed for the rapid detection of five types of anti-tuberculosis drug resistance, depending on the identification of common mutations in the* rpoB*,* katG*,* inhA*,* rrs*,* gyrA,* and* embB* genes. Relying on specific probes immobilized on nitrocellulose strips, GenoType MTBDR*plus* can detect drug resistance to RFP and INH, while the second version of GenoType MTBDR*sl* also enables the detection of mutations involved in resistance to injectable drugs, as well as resistance to FLQs. Evaluation studies of MTBDR*plus* and MTBDR*sl* have been conducted in different countries [14], but little research has been conducted in China. Hence, the present study aimed to evaluate the performance of GenoType MTBDR*plus* and GenoType MTBDR*sl* compared to conventional DST and to describe the patterns of drug resistance in a Chinese population.

## 2. Methods

### 2.1. Sample Collection

Sputum samples from newly diagnosed sputum smear-positive tuberculosis patients were collected in Jiangsu province, China, between May 2008 and December 2008. The samples were cultured and isolated on Lowenstein-Jensen (LJ) medium, followed by DST. Sputum smear microscopy testing and sputum culture were performed in the county-level laboratory, while the DST was performed at the provincial laboratory. The DNA of* M. tuberculosis* was extracted from the isolated culture and was used for the rapid detection of drug resistance by GenoType MTBDR*plus* and GenoType MTBDR*sl*. The researchers who performed the LPAs were blinded to the results of conventional DST. DNA sequencing was used to confirm genetic mutations and to explore the inconsistent and controversial results between conventional DST and the LPAs. We used the* M. tuberculosis* H37Rv strain as the control during the microbiological and genetic procedures.

### 2.2. Conventional DST

After strain isolation, DST was performed using the proportional method on LJ solid medium with critical concentrations of 40 *μ*g/mL for RFP, 0.2 *μ*g/mL for INH, 2.0 *μ*g/mL for OFX, 30.0 *μ*g/mL for Km, and 2 *μ*g/mL for EMB. The growth of colonies on the drug-containing plate was compared to the control plate as a proportion. If the bacterial growth on the medium with the specific drug was ≥1% greater than the control, the strain was declared resistant to the specific drug, and it was defined as sensitive when the growth rate was <1% greater than the control sample.

### 2.3. Genomic DNA Extraction

One loop of mycobacterial colonies on LJ medium was spun down and suspended in 200 *μ*L of TE buffer (10 mM Tris-HCl, 1 mM EDTA) in a 1.5 mL Eppendorf tube. Then, the mixture was incubated at 85°C for 30 minutes before it was centrifuged at 8000 rpm for 5 minutes. The supernatant layer containing DNA was collected and stored at −20°C until used.

### 2.4. GenoType MTBDR*plus* and GenoType MTBDR*sl* Testing

The GenoType MTBDR*plus* and GenoType MTBDR*sl* testing was performed according to the instructions supplied by the manufacturer (Hain Lifescience GmbH, Nehren, Germany). If any wild-type band was absent, or any mutation band was present, that particular strain was considered drug resistant. In contrast, if all of the wild-type bands were present and none of the mutation bands were present, that particular strain was considered susceptible.

### 2.5. PCR and DNA Sequencing

Fragments of* Rv0577* and* 16S rRNA* genes were amplified to identify the nontuberculous mycobacterial (NTM) strains, which were inconsistently judged by MTBDR*plus*, MTBDR*sl*, and conventional DST. To confirm the genetic mutations, the fragments of eight genes (*rpoB*,* katG*,* inhA*,* gyrA*,* gyrB*,* rrs*,* eis*, and* embB*) were amplified and sequenced with the primers listed in Table 1. PCR was conducted as follows: 94°C for 5 min, 35 cycles of denaturation at 94°C for 30 s, annealing at 65°C for 30 s, and elongation at 72°C for 1 min, followed by a final extension step for 10 min at 72°C. The PCR products were purified and sequenced.

### 2.6. Data Analysis

The data were entered using EpiData software, version 3.1 (Denmark), and were analyzed using STATA software, version 10.0 (StataCorp, College Station, TX, USA). Conventional DST was considered the gold standard for calculating the sensitivity, specificity, positive likelihood ratio (PLR), negative likelihood ratio (NLR), and agreement rate of the LPAs. The sequencing data were processed and analyzed by ProSeq software, version 3.0, and BioEdit software, version 7.1.9.

### 2.7. Ethical Consideration

The Institutional Review Board (IRB) of Nanjing Medical University approved the study. Written informed consent was obtained from all participants. The investigation was conducted according to the principles expressed in the Declaration of Helsinki.

## 3. Results

### 3.1. Strain Identification

We isolated 112 specimens circulating in Jiangsu province. Five strains were confirmed as NTM by both conventional DST and LPAs. For eight suspected NTM strains with inconsistent results, PCR was performed to amplify specific fragments of* 16s rRNA* and* Rv0577* genes. They were confirmed as NTM based on agarose gel electrophoresis and were excluded from subsequent analysis (Table 2).

### 3.2. Conventional DST

The most common drug-resistant patterns were RFP-R + INH-R + OFX-S + Km-S + EMB-S (24.24%) and RFP-R + INH-R + EMB-R + OFX-S + Km-S (14.14%). The proportions of drug-resistant strains for RFP, INH, OFX, Km, and EMB were 91.92%, 82.83%, 41.30%, 8.70%, and 51.52%, respectively. According to the conventional DST, there were five strains resistant to and one strain susceptible to all five drugs (Table 3).

### 3.3. Performance of GenoType MTBDR*plus* and GenoType MTBDR*sl*

The sensitivity, specificity, PLR, NLR, and Kappa values for RFP, INH, OFX, Km, and EMB using LPAs are shown in Table 4. Predominant patterns of mutation are listed in Table 5. Patterns of drug resistance detected by GenoType MTBDR*plus* and GenoType MTBDR*sl* in detail are displayed in Supplementary Table  1 (Supplementary Material available online at http://dx.doi.org/10.1155/2016/2064765).

For GenoType MTBDR*plus*, 95.96% (95/99) and 78.65% (70/89) had consistent phenotypic and genotypic results for RFP and INH, respectively. GenoType MTBDR*plus* yielded four false-positive results in RFP-susceptible strains. They showed omission of different* rpoB* wild-type bands (WT3 + WT4, WT6, WT7, and WT8). No false negative results for RFP were observed. Dual mutation (presence of* rpoB*MUT2A + MUT3) was observed in one strain. Three strains showed heteroresistance for RFP, with the appearance of both wild and mutation bands. GenoType MTBDR*plus* did not perform well for INH. Nine false-positive strains showed* inhA*MUT1 mutations, and 10 false negative strains did not show any omission of* katG*WT or* inhA*WT. No strain had* inhA*MUT2,* inhA*MUT3A, or* inhA*MUT3B mutations in our study. Notably, three strains (omission of both the* katG* control band and wild-type band) and seven strains (*inhA* control band was very weak) were identified as INH-invalid. Only one strain showed a mixed band pattern (*inhA*MUT1 presence + WT omission) while showing* katG*MUT1. No dual mutation was observed in the* katG* or* inhA* bands.

GenoType MTBDR*sl* correctly recognized 36 cases of OFX resistance in 38 strains, five cases of Km resistance in eight strains, and 30 cases of EMB resistance in 51 strains. Heteroresistance was common for OFX, while 15 (37.5%, 15/40) strains were obvious and six (15%, 6/40) were suspected. Thirteen of the strains had dual mutations. Regarding Km,* rrs*MUT1 was observed in all of the Km-resistant strains and* rrs*MUT2 was not observed in any strain. Three (50%) strains heteroresistant for Km presented with the* rrs*MUT1 band together with the* rrs*WT band. MUT1B was commonly observed in EMB-resistant strains. Two heteroresistant strains for EMB were detected by MTBDR*sl*, showing the presence of the bands of* embB*MUT1A + WT and* embB*MUT1B + WT.

### 3.4. Sequencing

Subsequent sequencing was performed in strains meeting the following criteria: (1) omission of both wild-type bands and mutation bands, indicating uncertain mutations; (2) mutation bands, wild-type bands, or gene locus control bands that were weak; (3) inconsistent results between phenotypes and genotypic assays; and (4) unavailable conventional DST results for OFX and Km. Supplementary Table  1 reveals the sequencing results. For the* rpoB* gene, codon 531 (47/95) was the most common mutation locus, followed by codon 526 (28/95) and codon 516 (11/95). Four false-positive strains identified by GenoType MTBDR*plus* were confirmed by sequencing to have mutations at codons 533, 526, 522, and 516 which were consistent with the target mutation regions of probes. Interestingly, two strains with the absence of the WT1 band for mutations were expected at 505–509. However, they were both observed by sequencing to have the mutation at codon 572. One RFP-resistant strain with the omission of both WT3 and WT8 was found to have an uncommon mutation at codon 515 (ATC→ACC). Sequencing of the* katG* and* inhA* (promoter region) genes confirmed mutations in all strains with the omission of both the wild and mutation bands and strains with weak bands. Of the three strains with omission of both the* katG* wild and mutation bands, in addition to codon* katG* 315, one was also found to have a mutation at codon* katG*317 and the other was at* inhA*-15 as determined by sequencing. Of four strains with omission of both the* inhA* wild-type band and mutation band, three strains were found to have a mutation at codon* katG*315 and one strain was found to have a mutation at* inhA*-34, rather than the target mutation of* inhA*-8. Notably, only one strain with omission of the* katG* locus control band was confirmed to have no mutation in the* katG* and* inhA* promoter region, while the other two strains failed in sequencing because of the quality of the samples. Of strains with weak* inhA* locus control bands, two strains were found to have mutations at* inhA*-26. Almost all of the strains with inconsistent INH resistance results were confirmed by sequencing to have concordant results with MTBDR*plus* except for one strain, which was found to be consistent with the conventional DST and to have a mutation at codon* katG*299. Sequencing confirmed mutations in strains with omission of both the wild and mutation bands or strains with weak bands in* gyrA*,* rrs*, and* embB*. One OFX-resistant strain with omission of both the* gyrA*WT2 and MUT bands was found to have mutations at codons* gyrA*90 and* gyrA*91, which were in the target mutation region (codons 89–93). Sequencing almost showed concordant results with GenoType MTBDR*sl* in strains with inconsistent resistance results. Two OFX-resistant strains with the* gyrA* wild-type band were sequenced, finding mutations at codons* gyrB*511 and* gyrB*422+435+450+473. Three of seven strains with unavailable DST results for OFX were found to have mutations at the target regions. One Km-susceptible strain was shown by sequencing to have concordant results and mutation loci with GenoType MTBDR*sl*. One of three Km-resistant strains was confirmed to have a mutation at* eis*-10, while the other two strains showed no mutations in the* rrs* and* eis* genes. One of seven strains with unavailable DST results for Km showed mutations at loci* rrs*1491, which was not covered by the target loci of GenoType MTBDR*sl*. Among strains with inconsistent resistance results for EMB, all of the four EMB-susceptible strains had mutations at codon* embB*306, and four of 21 EMB-resistant strains showed mutations at codons* embB*306,* embB*319,* embB* 407, and* embB* 410.

## 4. Discussion

GenoType MTBDR*plus* and GenoType MTBDR*sl* have already been in use in several countries, but they remain in the research stage in China, and information about their performance is of significance for future applications. In this study, we analyzed the drug resistance of RFP, INH, OFX, Km, and EMB by comparing LPA with phenotypic conventional DST in 99* M. tuberculosis* strains from Jiangsu Province, China. Subsequently, we sequenced strains with unclear results, unclear mutations, or inconsistent results.

In this study, the specificity for RFP and INH and the sensitivity for INH were much lower than studies conducted in Spain, Italy, South Africa, and Germany [15–19], while the results for OFX, Km, and EMB were similar to those of previous reports [20–27]. GenoType MTBDR*plus* and GenoType MTBDR*sl* showed high sensitivity for RFP and OFX, so it could be used in areas with high prevalence of drug resistance to detect potentially drug-resistant patients. Moreover, its high specificity for OFX could exclude OFX-susceptible patients in screening. However, the low sensitivity and specificity for Km and EMB have restricted the application of GenoType MTBDR*sl* for resistance to these two drugs. Our findings suggested that GenoType MTBDR*plus* and GenoType MTBDR*sl* could be used for early diagnosis and timely therapeutic instruction, while conventional DST can be used for confirmation, which requires several weeks.

The sensitivity for RFP resistance in this study (100%) was similar to that described in Spain (100%) [15] and Italy (100%) [16] but slightly higher than that reported in South Africa (98.95%) [18] and Germany (96.77%) [19]. Nevertheless, the specificity for RFP was much lower than that in the aforementioned studies, ranging from 95.45% to 100%. Four strains with false-positive results were confirmed by sequencing to have mutations in the* rpoB* gene. Two factors might have contributed to these inconsistent results. First, the sample size of RFP-susceptible strains was small, and only eight RFP-susceptible strains were recruited for this study. In other words, the proportion of RFP-resistant strains was much higher than that in previous studies [15–19], thus incurring sample selection bias. Second, conventional DST, as the gold standard, was not always perfect. Conventional DST for RFP was not absolutely as accurate and reliable as we expected because its performance was not as straightforward in the rounds of proficiency testing among the supranational TB reference laboratories (SRL) [28]. It showed highly inconsistent results between these top laboratories in detecting strains with specific mutations, that is, the “disputed” mutations [29]. The MICs of stains with these “disputed” mutations could be less than the conventional critical concentrations [30], leading to a “susceptible” result. The specificity of molecular detection assay might be underevaluated because of the limitations of conventional DST [31]. In this study, we observed that the mutation frequency of* rpoB*S531L in RFP-resistant strains detected by GenoType MTBDR*plus* was 42.11%, which was lower than that in studies conducted in Colombia (64%) and Spain (72.2%) [16, 32]. Mutations confirmed by sequencing were almost all located in the target mutation region of GenoType MTBDR*plus* except for the mutation of* rpoB*I572T in two strains with omission of WT1, for which the target mutation region was codons 505–509. There were many mutations in the rpoB gene detected by sequencing, but their roles in RFP resistance require more studies to confirm.

The main reasons for the low sensitivity and specificity in detecting INH resistance might be similar to those for RFP, which include sample selection bias and the accuracy of conventional DST. In addition, the mechanism of INH resistance has not been entirely clear, and it might have contributed to the low sensitivity for INH. The proportion of INH-resistant strains in our study was much higher than that in previous studies (26.8%–68%) [15–19]. According to the Kim summary, at the concentration of 0.2 *μ*g/mL for INH, the 1% critically resistant proportion could likely distinguish between susceptible and resistant strains, showing a discrimination power of 77.1% [33]. Moreover, even in different laboratories using the same methods, the most reasonable criteria for resistance could be different [33]. Hazbon et al. found that approximately 10–15% of low-level INH-resistant strains did not have mutations in* katG* or* inhA* [34]. Heym et al. also found mutations in the promoter region of ahpC in INH-resistant strains [35]. All of these findings supported that INH resistance might be due to a new mechanism. The limited numbers of probes in GenoType MTBDR*plus* restricted its detection of all mutation loci, which might also have decreased its sensitivity. Among three strains with omission of the* katG* control band and WT band, sequencing did not discover any mutations in one strain and failed in the other two strains. The sequencing result was consistent with DST but discrepant with the manufacturer's instructions, which classified this situation as resistance to INH. We should be more cautious about similar situations and repeat the experiments to confirm the results.

The high sensitivity, specificity, and Kappa value of GenoType MTBDR*sl* for OFX indicated the high consistency between this rapid detection assay and conventional DST. The common mutations identified by GenoType MTBDR*sl* or sequencing in OFX-resistant strains were* gyrA*A91V and D94G. Differing from previous studies in which the D94G mutation was much more common than A91V, the frequency of these two mutations was close to each other in our study [20, 26, 36, 37]. Heteroresistance of OFX detected by GenoType MTBDR*sl* in our study was higher than previous reports [20, 26, 36, 37]. It is notable that six strains with weak mutation bands which were suspected to have heteroresistance were confirmed to have no heteroresistance by sequencing. Moreover, some of the 15 heteroresistant strains were confirmed to have no heteroresistance by sequencing. These sequenced strains were observed to have mutations at codons 94 and 90 except for one strain, which had a mutation at codon 95, which was considered not to take part in fluoroquinolone resistance [8]. We suspected that mutations at codons 94 and 90 may easily lead to a detection result of heteroresistance by GenoType MTBDR*sl*. Though we found mutations at* gyrB*511 and* gyrB*422 in two OFX-susceptible strains, we could not confirm that the mutations were associated with OFX resistance because the mutation locus in* gyrB* gene in OFX-resistant strains varied greatly in previous studies [38–40].

The Km-resistant strains judged by GenoType MTBDR*sl* were all confirmed to have mutations of* rrs*A1401G, but one Km-susceptible strain was also found to have an* rrs*A1401G mutation. It was reported that the A1401G mutation in the* rrs* gene was associated with drug resistance to Km and Am [41]. Moreover, the mutation at position 1401 might be a better marker for Am resistance than for Km resistance [20]. The mutation in the* eis* gene promoter region was reported to be associated with Km resistance [42]. In our study, one of three Km-resistant strains without mutations in the* rrs* gene detected by GenoType MTBDR*sl* was confirmed to have a mutation at* eis*-10.

The low detection ability of EMB resistance indicated that the molecular basis of EMB resistance in GenoType MTBDR*sl* was insufficient, although* embB*306 was common in the EMB-resistant strains [43, 44]. In addition to M306I and M306V, some rare mutations—Y319C, G407A, and A410P—were also observed in this study. There might also exist some mutations in the* embA* or* embC* gene related to EMB resistance, rather than mutations in* embB* [45].

In conclusion, GenoType MTBDR*plus* and GenoType MTBDR*sl* could be applied for the rapid detection of drug resistance to RFP and OFX. However, the role of GenoType MTBDR*plus* for INH resistance detection was not confirmed because the current results were different from previous reports. It cannot be widely applied until further validation in China. In addition, because the mechanism of Km and EMB resistance was not completely identified, GenoType MTBDR*sl* for detecting resistance to Km and EMB is not currently suitable for clinical applications. The correlation between uncommon mutations identified in this study and drug resistance must be confirmed in the future.

## Supplementary Material


Supplementary Table 1 showed the pattern of drug resistance detected by GenoType MTBDR*plus* and MTBDR*sl* and the mutations found in *rpoB*, *katG*, *inhA*, *gyrA*, *gyrB*, *rrs*, *eis*, and *embB* gene by sequencing. Though some strains showed the same drug resistant results by GenoType MTBDR*plus* and MTBDR*sl*, the bands pattern on the detection strip and their target mutation locus were various, which were all listed in Supplementary Table 1 together with the conventional DST results. Sequencing analysis revealed not only the mutations consistent with mutation target of GenoType MTBDR*plus* and MTBDR*sl*, but also some uncommon mutations and mutation combinations.

## Figures and Tables

**Table 1:** PCR and DNA sequencing primers.

Locus	Primer	Sequence (5′ to 3′)	Size (bp)	Position	Product (bp)
Rv0577	Rv0577-f	ATGCCCAAGAGAAGCGAATACAGGCAA	27	671166⋯671192	786
	Rv0577-r	CTATTGCTGCGGTGCGGGCTTCAA	24	671951⋯671928	786

16s rRNA	16SrRNA-f	ACGGTGGGTACTAGGTGTGGGTTTC	25	1472650–674	543
	16SrRNA-r	TCTGCGATTACTAGCGACTCCGACTTCA	28	1473192–165	543

*rpoB*	rpoB-f	CTTGCACGAGGGTCAGACCA	20	760829⋯760848	543
	rpoB-r	ATCTCGTCGCTAACCACGCC	20	761371⋯761352	543

*inhA* (promoter)	inhA-f	TGCCCAGAAAGGGATCCGTCATG	23	2154886⋯2154905	455
	inhA-r	ATGAGGAATGCGTCCGCGGA	20	2155340⋯2155321	455

*katG*	katG-f	AACGACGTCGAAACAGCGGC	20	2154886⋯2154905	455
	katG-r	GCGAACTCGTCGGCCAATTC	20	2155340⋯2155321	455

*gyrA*	gyrA-f	CCCTGCGTTCGATTGCAAAC	20	7273⋯7292	423
	gyrA-r	CTTCGGTGTACCTCATCGCC	20	7695⋯7676	423

*embB*	embB-f	CTGACCGACGCCGTGGTGATAT	22	4247345⋯4247366	490
	embB-r	TGAATGCGGCGGTAACGACG	20	4247834⋯4247815	490

*rrs*	rrs-r	GTCCGAGTGTTGCCTCAGG	19	1473518⋯1473500	516
	rrs-f	GTCAACTCGGAGGAAGGTGG	20	1473003⋯1473022	516

*eis*	eis-r	GCGTAACGTCACGGCGAAATTC	22	2715477⋯2715456	567
	eis-f	GTCAGCTCATGCAAGGTG	18	2714911⋯2714928	567

gyrB	gyrB-f	AAGACCAAGTTGGGCAACAC	20	6353⋯6372	609
	gyrB-r	CTGCCACTTGAGTTTGTACA	20	6961⋯6942	609

**Table 2:** NTM detected by conventional DST and GenoType MTBDR*plus* and GenoType MTBDR*sl*.

Strain number	Conventional DST						MTBDR*plus*			MTBDR*sl*			
	Strain	RFP	INH	OFX	Km	EMB	strain	RFP	INH	Strain	OFX	Km	EMB
966	MTB	R	R	R	S	R	NTM						
1246	NTM	—	—	—	—	—	MTB	R	R	MTB	S	S	R
1378	NTM	—	—	—	—	—	MTB	R	R	MTB	S	S	S
1491	NTM	—	—	—	—	—	MTB	R	R	MTB	S	S	R
2052	NTM	—	—	—	—	—	MTB	S	S	MTB	S	S	S
1538	MTB	R	S	S	S	R	NTM	—	—	—	—	—	—
1581	MTB	S	R	R	R	NA	NTM	—	—	—	—	—	—
1782	MTB	R	R	NA	NA	R	NTM	—	—	—	—	—	—
1545	NTM	—	—	—	—	—	NTM	—	—	—	—	—	—
1897	NTM	—	—	—	—	—	NTM	—	—	—	—	—	—
1901	NTM	—	—	—	—	—	NTM	—	—	—	—	—	—
1902	NTM	—	—	—	—	—	NTM	—	—	—	—	—	—
1939	NTM	—	—	—	—	—	NTM	—	—	—	—	—	—

R: resistant; S: sensitive; NA: not available.

**Table 3:** Drug resistance patterns detected by conventional DST in 99 strains.

Number (%) of strains	RFP	INH	OFX	KAN	EMB
6 (6.06)	R	R	NA	NA	R
1 (1.01)	R	R	NA	NA	S
5 (5.05)	R	R	R	R	R
1 (1.01)	R	R	R	R	S
16 (16.16)	R	R	R	S	R
11 (11.11)	R	R	R	S	S
1 (1.01)	R	R	S	R	R
1 (1.01)	R	R	S	R	S
14 (14.14)	R	R	S	S	R
24 (24.24)	R	R	S	S	S
1 (1.01)	R	S	R	S	R
2 (2.02)	R	S	R	S	S
3 (3.03)	R	S	S	S	R
5 (5.05)	R	S	S	S	S
1 (1.01)	S	R	S	S	R
1 (1.01)	S	R	S	S	S
1 (1.01)	S	S	R	S	R
1 (1.01)	S	S	R	S	S
3 (3.03)	S	S	S	S	R
1 (1.01)	S	S	S	S	S

R: resistant; S: sensitive; NA: not available.

**Table 4:** Performance of GenoType MTBDR*plus* and GenoType MTBDR*sl* according to the conventional DST.

Conventional DST (*n*)	GenoType MTBDR*plus* and GenoType MTBDR*sl*									
	R	S	INV	Se (%)	Sp (%)	PLR	NLR	Agr (%)	Kappa	*P* ^#^
*RFP*										
R (91)	91	0	0							
S (8)	4	4	0							
Total (99)	95	4	0	100	50	2	0	95.96	0.65	<0.001
*INH*										
R (82)	62	10	10^*∗*^							
S (17)	9	8	0							
Total (99)	71	18	10	86.11	47.06	1.63	0.30	78.65	0.32	0.001
*OFX*										
R (38)	36	2	0							
S (54)	4	50	0							
NA (7)	3	4	0	94.74	92.59	12.79	0.06	93.48	0.87	<0.001
Total (99)	43	56	0							
*Km*										
R (8)	5	3	0							
S (84)	1	83	0							
NA (7)	2	5	0	62.50	98.81	52.50	0.38	95.65	0.69	<0.001
Total (99)	8	86	0							
*EMB*										
R (51)	30	21	0							
S (48)	4	44	0							
Total (99)	34	65	0	58.82	91.67	7.06	0.45	74.75	0.50	<0.001

R: resistant; S: sensitive; NA: not available; Se: sensitivity; Sp: specificity; INV: invalid; PLR: positive likelihood ratio; NLR: negative likelihood ratio; Agr: agreement.

^*∗*^Seven strains with very weak *inhA* locus control bands and 3 strains without *katG* control bands were identified as invalid results.

^*#*^Significant test for Kappa.

**Table 5:** Predominant mutation patterns for RFP, INH, OFX, Km, and EMB.

Drug	Predominant mutation patterns	Number of strains
RFP	*rpoB*WT8 omission + MUT3 appearance	40

INH	*katG*WT omission + MUT1 appearance	54
	*inhA*WT1 omission + MUT1 appearance	12

OFX	*gyrA*WT3 omission + MUT3C appearance	7

Km	*rrs*MUT1 appearance	8

EMB	*embB*WT omission + MUT1B appearance	15
