# Supplementary material for: Evaluation of MTBDRplus and MTBDRsl in Detecting Drug-Resistant Tuberculosis in a Chinese Population
Source: Dis Markers. 2016 Jul 25;2016:2064765. doi: 10.1155/2016/2064765 (PMC4976146; doi:10.1155/2016/2064765)
Supplement: Supplementary file 1 — Supplementary Table 1 showed the pattern of drug resistance detected by GenoType MTBDRplus and MTBDRsl and the mutations found in rpoB, katG, inhA, gyrA, gyrB, rrs, eis, and embB gene by sequencing. Though some strains showed the same drug resistant results by GenoType MTBDRplus and MTBDRsl, the bands pattern on the detection strip and their target mutation locus were various, which were all listed in Supplementary Table 1 together with the conventional DST results. Sequencing analysis revealed not only the mutations consistent with mutation target of GenoType MTBDRplus and MTBDRsl, but also some uncommon mutations and mutation combinations. [file 2064765.f1.pdf]

Supplemental Table 1. Patterns of drug resistance by GenoType MTBDR*plus* and MTBDR*sl* and sequencing analysis

Drug	Band on the strip	Target mutation	Strain No.	Conventional DST (Strain No.)	Sequencing (Strain No.)	Mutation by sequencing（amino acid）（Strain No.）
**RFP**						
	rpoB△WT8, MUT3	S531L	40	R (40)		
	rpoB△WT8	530-533	8	R (7)	√ (7)	*rpoB* TCG(S) 531 ATG(M) (1)
						rpoB TCG(S) 531 TGG(W) + *rpoB* GAG(E) 504 GCG(A) (1)
						rpoB TCG(S) 531 TGG(W) (2)
						rpoB TCG(S) 531 CAG(Q) (1)
						rpoB CTG(L) 533 CCG(P) + *rpoB* GAC(D) 516 GCC(A) (1)
						rpoB CTG(L) 533 CCC(P) + *rpoB* AAC(N) 518 AGC(S) (1)
				S (1)	√ (1)	*rpoB* CTG(L) 533 CCG(P) (1)
	rpoB△WT7,△WT8	526-533	2	R (2)	√ (2)	*rpoB* TCG(S) 531 GCG(A) + *rpoB* CAC(H) 526 CTC(L) (1)
						rpoB CAC(H) 526 AAC(N) (1)
	rpoB△WT7,MUT2B	H526D	6	R (6)		
	rpoB△WT7,MUT2A	H526Y	8	R (8)		
	rpoB△WT7,MUT1	D516V	1	R (1)		
	rpoB△WT8,MUT3	S531L	40	R (40)		
	rpoB△WT8	530-533	8	R (7)	√ (7)	*rpoB* TCG(S) 531 ATG(M) (1)
						rpoB TCG(S) 531 TGG(W) + *rpoB* GAG(E)

					rpoB TCG(S) 531 TGG(W) (2)
					rpoB TCG(S) 531 CAG(Q) (1)
					rpoB CTG(L) 533 CCG(P) + *rpoB* GAC(D)
					rpoB CTG(L) 533 CCC(P) + *rpoB* AAC(N)
			S (1)	√ (1)	*rpoB* CTG(L) 533 CCG(P) (1)
*rpoB*ΔWT7,ΔWT8	526-533	2	R (2)	√ (2)	*rpoB* TCG(S) 531 GCG(A) + *rpoB* CAC(H)
					rpoB CAC(H) 526 AAC(N) (1)
*rpoB*ΔWT7,MUT2B	H526D	6	R (6)		
*rpoB*ΔWT7,MUT2A	H526Y	8	R (8)		
*rpoB*ΔWT7,MUT1	D516V	1	R (1)		
*rpoB*ΔWT7,(MUT2B)	526-529	3	R (3)	√ (3)	*rpoB* CAC(H) 526 GAC(D) + *rpoB* GAA(E)
					rpoB CAC(H) 526 GAC(D) (2)
*rpoB*ΔWT7,(MUT2A)	526-529	2	R (2)	√ (2)	*rpoB* CAC(H) 526 TAC(Y) (2)
*rpoB*ΔWT7	526-529	6	R (5)	√ (5)	*rpoB* CAC(H) 526 CGC(R) (2)
					rpoB CAC(H) 526 GAC(D) (2)
					rpoB CAC(H) 526 CTC(L) (1)
			S (1)	√ (1)	*rpoB* CAC(H) 526 CTC(L) (1)
*rpoB*ΔWT6	518-525	3	R (2)	√ (2)	*rpoB* TCG(S) 522 TTG(L) (2)
			S (1)	√ (1)	*rpoB* TCG(S) 522 TTG(L) (1)
*rpoB*ΔWT3,ΔWT8	513-517,	1	R (1)	√ (1)	*rpoB* ATG(M) 515 CTG(L) + *rpoB* CTG(L)
*rpoB*ΔWT3,ΔWT4,ΔWT8	513-519,	3	R (3)	√ (3)	*rpoB* GAC(D) 516 GGC(G) + *rpoB* CTG(L)
					rpoB GAC(D) 516 GCC(A) + *rpoB* CTG(L)
*rpoB*ΔWT3,ΔWT4,MUT1	D516V	2	R (2)		
*rpoB*ΔWT3,ΔWT4	513-519	2	R (1)	√ (1)	*rpoB* GAC(D) 516 TAC(Y) + *rpoB* CCC(P)
			S (1)	√ (1)	*rpoB* GAC(D) 516 GGC(G) (1)

*rpoB*ΔWT2,ΔWT7	510-513,	1	R (1)	√ (1)	*rpoB* CAC(H) 526 CGC(R) + *rpoB* AGC(S)
*rpoB*ΔWT2,ΔWT3	510-517	1	R (1)	√ (1)	*rpoB* CAA(Q) 513 CTA(L) (1)
*rpoB*ΔWT1	505-509	2	R (2)	√ (2)	*rpoB* ATC(I) 572 ACC(T) (2)
*rpoB*ΔWT,MUT2B	H526D	1	R (1)		
*rpoB*MUT2A,MUT3	H526Y,	1	R (1)		
*rpoB*MUT1	D516V	2	R (2)		
*rpoB*WT		4	S (4)		
**INH**					
*katG*ΔWT,MUT2,(MUT1)	S315T	1	R (1)	√ (1)	*katG* AGC(S) 315 ACA(T)(1)
*katG*ΔWT,MUT1	S315T	54	R (53)		
			S (1)	√ (1)	*inhA* C -15 T (1)
*katG*ΔWT,MUT2	S315T	1	R (1)		
*katG*ΔWT	315	3	R (3)	√ (3)	*katG* AGC(S) 315 AAC(N) (1)
					katG AGC(S) 315 AAC(N) + *katG* ATC(I)
					katG AGC(S) 315 AAC(N) + *inhA* C -15 T (1)
Δ*katG*ΔWT	315	3	R (3)	√ (3)	*katG, inhA* NM (1)
					NA (2)
*katG*WT		37	R (21)		
			S (16)		
*inhA*ΔWT1,(MUT1)	-15	1	R (1)	√ (1)	*inhA* C -15 T (1)
*inhA*ΔWT2	-8	4	R (4)	√ (4)	*katG* AGC(S) 315 AAC(N) (1)
					katG AGC(S) 315 ACC(T) (2)
					inhA C-34 T (1)
*inhA*MUT1	C-15T	1	R (1)		
*inhA*WT		74	R (66)		

				S (8)		
	inhA∆WT1,MUT1	C-15T	12	R (3)		
				S (9)	√ (9)	*inhA* C -15 T (9)
	(*inhA*)*inhA*WT		4	R (4)	√ (4)	*katG, inhA* NM (4)
	(*inhA*)(*inhA*WT2)*inhA*WT1	-16	3	R (3)	√ (3)	*inhA* C -26 T (2)
						katG, inhA NM (1)
	*katG*WT+ *inhA*WT[*]		18	R (10)	√ (10)	*katG* GGC(G) 299 GTC(V) (1)
**OFX**						*katG, inhA* NM (9)
	gyrA∆WT3,MUT1,MUT3B	A90V,	3	R (3)		
	gyrA∆WT3,MUT3D,(MUT1)	A90V,	1	R (1)	√ (1)	*gyrA* GAC(D) 94 CAC(H) (1)
	gyrA∆WT3,MUT3A	D94A	2	R (1)	√ (1)	
				S (1)		*gyrA* GAC(D) 94 GCC(A) (1)
	gyrA∆WT3,MUT3B	D94N	2	R (2)		
	gyrA∆WT3,MUT3C	D94G	7	R (5)		
	gyrA∆WT3,MUT1	A90V	2	R (2)		
	gyrA∆WT2,MUT1	A90V	3	R (2)		
				S (1)	√ (1)	*gyrA* GCG(A) 90 GTG(V) (1)
**OFX**						
	gyrA∆WT2,MUT2	S91P	1	R (1)		
	gyrA∆WT2	89-93	1	R (1)	√ (1)	*gyrA* GCG(A) 90 GTG(V) + *gyrA* TCG(S) 91
	*gyrA*MUT3A,(MUT1,WT3)	(A90V),	1	R (1)	√ (1)	*gyrA* GAC(D) 94 GCC(A) (1)
	*gyrA*MUT3A	D94A	3	R (3)		
	*gyrA*MUT3C,(MUT1,WT3)	(A90V),	3	R (2)	√ (2)	*gyrA* GAC(D) 94 GGC(G) (2)
		D94G		---† (1)	√ (1)	*gyrA* GAC(D) 94 GGC(G) (1)
	*gyrA*MUT3C	D94G	2	R (2)		

	*gyrA*MUT1,MUT3C,(WT3)	A90V,	2	R (2)	√ (2)	*gyrA* GAC(D) 94 GGC(G) (2)
	*gyrA*MUT1,MUT3C	A90V,	2	R (2)		
	*gyrA*MUT1,(MUT3C)	A90V,	1	S (1)	√ (1)	*gyrA*, *gyrB* NM (1)
	*gyrA*MUT1,(WT2)	A90V	1	R (1)	√ (1)	*gyrA* GCG(A) 90 GTG(V) (1)
	*gyrA*MUT1	A90V	6	R (5)		
				S (1)	√ (1)	*gyrA* GCG(A) 90 GTG(V) (1)
	*gyrA*WT		56	R (2)	√ (2)	*gyrB* ACC(T) 511 AAC(N) (1)
						gyrB GTG(V) 422 GGG(G) + *gyrB* CGA(R)
				S (50)		
				---† (4)	√ (4)	*gyrA*, *gyrB* NM (4)
**Km**						
	rrs∆WT1,MUT1	A1401G	4	R (3)		
				---† (1)	√ (1)	*rrs*, *eis* NM (1)
	*rrs*MUT1,(WT1)	A1401G	1	R (1)	√ (1)	*rrs* A 1401 G (1)
	*rrs*MUT1	A1401G	3	R (1)		
				S (1)	√ (1)	*rrs* A 1401 G (1)
				---† (1)	√ (1)	*rrs* T 1491 A (1)
	*rrs*WT		89	R (3)	√ (3)	*rrs*, *eis* NM (2)
						eis G -10 A（1）
				S (81)		
				---† (5)	√ (5)	*rrs*, *eis* NM (5)
	rrs (WT)		2	S (2)	√ (2)	*rrs* A 1401 G (1)
						rrs, *eis* NM (1)
**EMB**						
	embB∆WT,MUT1A	M306I	11	R (10)		

			S (1)	√ (1)	*embB* ATG(M) 306 ATA(I) (1)
embB∆WT,MUT1B	M306V	15	R (13)		
			S (2)	√ (2)	*embB* ATG(M) 306 GTG(V) (2)
embB∆WT	306	6	R (5)	√ (5)	*embB* ATG(M) 306 ATT(I) (2)
					embB ATG(M) 306 ATC(I) (2)
					embB ATG(M) 306 CTG(L) (1)
			S (1)	√ (1)	*embB* ATG(M) 306 ATT(I) (1)
embB MUT1A	M306I	1	R (1)		
embB MUT1B	M306V	1	R (1)		
embB WT		65	R (21)	√ (21)	*embB* ATG(M) 306 ATC(I) (1)
					embB TAT(Y) 319 TGT(I) (1)
					embB GGC(G) 407 GCC(A) (1)
					embB GCG(A) 410 CCG(P) (1)
					embB NM (15)
					NA (2)
			S (44)		

WT, presence of all wild-type bands for specific genes; ∆WT, omission of wild-type band; MUT, presence of specific mutation band; (WT) or (MUT), weak band; ∆*katG*, omission of *katG locus control band*; (*inhA*), *inhA* locus control band was very weak

R=resistant, S=sensitive, NA=not available, NM= no mutation

[*] To indicate INH resistance by reading both *katG* and *inhA* bands

† Conventional DST result was not available.
